# Phase 2 randomised placebo-controlled trial of spironolactone and dexamethasone versus dexamethasone in COVID-19 hospitalised patients in Delhi

**DOI:** 10.1186/s12879-023-08286-w

**Published:** 2023-05-15

**Authors:** Bharti Wadhwa, Vikas Malhotra, Sukhyanti Kerai, Farah Husain, Nalini Bala Pandey, Kirti N. Saxena, Vinay Singh, Tom M. Quinn, Feng Li, Erin Gaughan, Manu Shankar-Hari, Bethany Mills, Jean Antonelli, Annya Bruce, Keith Finlayson, Anne Moore, Kevin Dhaliwal, Christopher Edwards

**Affiliations:** 1 Department of Anaesthesia, Maulana Azad Medical College, New Delhi, India; 2 Department of ENT & Head and Neck Surgery, Maulana Azad Medical College & Associated Hospitals, New Delhi, India; 3 Centre for Inflammation Research, The Queen’s Medical Research Institute, BioQuarter, The University of Edinburgh, Edinburgh, EH16 4TJ UK; 4 Royal Infirmary of Edinburgh, BioQuarter, Little France, Edinburgh, EH16 4SA UK; 5 Imperial College, Hammersmith Campus, Du Cane Road, London, W12 0NN UK

**Keywords:** COVID-19, Spironolactone, Infectious diseases, Respiratory medicine

## Abstract

**Background:**

In this phase 2 randomised placebo-controlled clinical trial in patients with COVID-19, we hypothesised that blocking mineralocorticoid receptors using a combination of dexamethasone to suppress cortisol secretion and spironolactone is safe and may reduce illness severity.

**Methods:**

Hospitalised patients with confirmed COVID-19 were randomly allocated to low dose oral spironolactone (50 mg day 1, then 25 mg once daily for 21 days) or standard of care in a 2:1 ratio. Both groups received dexamethasone 6 mg daily for 10 days. Group allocation was blinded to the patient and research team. Primary outcomes were time to recovery, defined as the number of days until patients achieved WHO Ordinal Scale (OS) category ≤ 3, and the effect of spironolactone on aldosterone, D-dimer, angiotensin II and Von Willebrand Factor (VWF).

**Results:**

One hundred twenty patients with PCR confirmed COVID were recruited in Delhi from 01 February to 30 April 2021. 74 were randomly assigned to spironolactone and dexamethasone (SpiroDex), and 46 to dexamethasone alone (Dex). There was no significant difference in the time to recovery between SpiroDex and Dex groups (SpiroDex median 4.5 days, Dex median 5.5 days, *p* = 0.055). SpiroDex patients had significantly lower D-dimer levels on days 4 and 7 (day 7 mean D-dimer: SpiroDex 1.15 µg/mL, Dex 3.15 µg/mL, *p* = 0.0004) and aldosterone at day 7 (SpiroDex 6.8 ng/dL, Dex 14.52 ng/dL, *p* = 0.0075). There was no difference in VWF or angiotensin II levels between groups. For secondary outcomes, SpiroDex patients had a significantly greater number of oxygen free days and reached oxygen freedom sooner than the Dex group. Cough scores were no different during the acute illness, however the SpiroDex group had lower scores at day 28. There was no difference in corticosteroid levels between groups. There was no increase in adverse events in patients receiving SpiroDex.

**Conclusion:**

Low dose oral spironolactone in addition to dexamethasone was safe and reduced D-dimer and aldosterone. Time to recovery was not significantly reduced. Phase 3 randomised controlled trials with spironolactone and dexamethasone should be considered.

**Trial registration:**

The trial was registered on the Clinical Trials Registry of India TRI: CTRI/2021/03/031721, reference: REF/2021/03/041472. Registered on 04/03/2021.

**Supplementary Information:**

The online version contains supplementary material available at 10.1186/s12879-023-08286-w.

## Background

Most individuals infected with severe acute respiratory syndrome coronavirus-1 (SARS-CoV-2) experience mild symptoms, however the progression to severe COVID-19 is characterised by multi-organ failure, and a high risk of death [1, 2]. Identifying treatments to prevent clinical deterioration is imperative.

Early variants of SARS-CoV-2 required the Angiotensin Converting Enzyme 2 (ACE2) receptor, and transmembrane serine protease 2 (TMPRSS2) on the cell membrane to facilitate cell entry [3, 4]. Transcription of the TMPRSS2 gene is enhanced by androgens through androgen receptors (AR) [5], and anti-androgen treatments such as enzalutamide have been shown to reduce cellular entry of SARS-CoV-2 into human lung cells in in vitro studies[6]. The omicron variants appear not to be dependent on TMPRSS2. Down regulation of the ACE2 receptor as a consequence of viral infection may result in the loss of angiotensin II conversion to angiotensin [7]. The resultant high levels of unopposed angiotensin II, stimulates NADPH oxidase with consequent elevation of Reactive Oxygen Species (ROS). This results in the loss of the specificity of the Mineralocorticoid Receptor (MR), enabling stimulation by cortisol and aldosterone [8]. As the levels of cortisol are about 100 fold higher than aldosterone we hypothesised that spironolactone (which is a MR antagonist with anti-androgen and anti-inflammatory effects) in combination with dexamethasone, which suppresses cortisol secretion, could reduce time to recovery and severity of COVID-19 disease in hospitalised patients [6, 8–10]. This hypothesis is supported by results from retrospective and prospective non-randomised open-label studies of MR blockade in COVID-19 patients [8, 11, 12].

In this context, we report a phase 2 randomised placebo-controlled trial (RCT) comparing the addition of low dose spironolactone and dexamethasone (SpiroDex) to standard of care with dexamethasone (Dex) in a hospital setting in Delhi, India.

## Methods

### Trial design and patients

The trial was a single centre RCT. Patients were recruited between 01 February to 30 April 2021 from Maulana Azad Medical College and Lok Nayak Hospital, New Delhi. Patients were recruited if they had positive nasopharyngeal SARS-CoV-2 polymerase chain reaction (PCR) and/or had chest radiographic changes consistent with COVID-19 pneumonia, with an additional oxygen requirement (oxygen saturations (SpO2) < 94% on room air but maintaining SpO2 of ≥ 94% on supplemental oxygen by mask or nasal prongs). Exclusion criteria were: participation in another clinical trial of an investigational medicinal product (IMP); mechanical ventilation; known hypersensitivity to the IMP; significant electrolyte disturbance (Hyperkalaemia: K^+^  > 5.5 mmol/L); receiving potassium sparing diuretics that could not be reasonably withheld; acute renal insufficiency; if in the investigator’s opinion the patient was unwilling or unable to comply with drug administration plan, required laboratory tests or other trial procedures; pregnancy or lactating, or if the patient was on the following drugs: ACE inhibitors, amiloride, hydrocortisone, prednisolone, methylprednisolone, triamterene.

A sample size calculation was based on a primary biomarker endpoint (D-dimer: selected for its association with poor Covid-19 outcomes [13]) and powered to show a 60% change in treatment group compared to controls. Accepting a type 1 error rate of 0.05 the study required 108 patients at a 2 to 1 allocation providing 80% power with a 20% drop out rate. For study feasibility reasons a total of 120 patients was chosen.

### Randomisation and blinding

2:1 (SpiroDex:Dex) randomisation was performed utilising a coin toss method to achieve the desired probability. Multiple coin flips per patient (maximum of 6 flips per patient, or until ≥ 3 heads were recorded) were used to achieve a 2:1 randomisation. Further information on this method of randomisation is available in the supplementary information. After randomisation the group allocation was concealed in an opaque sealed envelope which was opened prior to the initiation of treatment. The group allocation was known only to the treating non-trial physician. The patients and the trial investigators recording the trial parameters were blinded to group allocation. Blank pills were used as the placebo pills.

### Interventions

Patients randomised to the SpiroDex group received oral spironolactone 50 mg once daily (as two tablets of 25 mg) on day 1, followed by 25 mg once daily until day 21. All patients received standard of care which included supportive measures for SARS-CoV-2 and approved therapies at the time as per Government of India guidelines (which included remdesivir for 26.1% of standard of care patients and 23.0% of patients within the SpiroDex group). Dexamethasone was given from day one at a dose of 3 mg twice daily for ten days. The control arm received two placebo tablets on day one, followed by one placebo tablet daily until day 21.

In the event of hospital discharge, dexamethasone was continued until day 10 in both groups, as per treatment guidelines. Spironolactone 25 mg was continued for a total period of 21 days in the trial group. Placebo tablets were continued until day 21 in the control arm. All patients were followed up with a telephone call at day 28 from the day of enrolment.

If a National Early Warning Score (NEWS2) score [14] became > 7 or WHO ordinal scale (WHO OS) [15] > 4 (hospitalised severe disease), the patient was withdrawn due to clinical deterioration and documented as having met the endpoint of clinical deterioration. No further clinical data was recorded thereafter.

### Clinical and laboratory monitoring

Baseline investigations were performed including full blood count, blood sugar, liver function tests, blood urea, serum creatinine, serum electrolytes, D-dimer, electrocardiogram (ECG), and chest X-ray (CXR). All patients were observed, and vital signs monitored daily (blood pressure, heart rate, temperature, oxygen saturation (SpO2), respiratory rate, NEWS2 score). The change in oxygen saturation to fraction of inspired oxygen ratio (SpO2/FiO2) was measured daily from the start of the trial until day 10 or hospital discharge. The duration of total hospital stay as well as the duration of oxygen use, and the time in days taken to achieve an SpO2 of ≥ 94% on room air were recorded in both groups. Self-reported cough symptom scores were recorded for all in-patients using an ordinal scale until day 10 or until patient discharge, and again at day 28 by follow-up phone call.

### Outcomes

The primary outcomes were time to recovery, (as defined as the first day after enrolment, where the patient met the criteria of WHO OS score ≤ 3 (hospitalised but no oxygen therapy)); this was recorded until hospital discharge or day 10 after enrolment in the study. Primary plasma biomarkers measured were circulating VWF, angiotensin II, aldosterone, and D-dimer levels taken at baseline, day 4 and day 7.

The secondary outcomes were to study the effect of spironolactone on the patient’s cough (as assessed by daily cough scores from baseline at enrolment until day 10, and a follow up cough score recorded via telephone call at day 28); to determine the duration (in days) of oxygen use and oxygen-free days (from baseline at enrolment until day 10 or hospital discharge, whichever was first); and to determine the effect of spironolactone and dexamethasone on plasma corticosteroid levels as a measure of suppression of the production of cortisol and blockade of the MR (taken at baseline, day 4 and day 7).

### Blood biomarker measurement

Patient serum was drawn on day 1, 4 and 7 to measure angiotensin II, cortisone, cortisol and aldosterone levels. Patient citrated plasma was used to measure VWF and D-Dimer. All assays were performed according to the manufacturer’s instructions. Concentrations were calculated against standard curves and used for statistical analysis. Blood glucose was measured by glucometer (Glucocare Sense, RMD Mediaids ltd, Haryana, India) and sodium, urea and potassium were measured as part of routine clinical care.

The circulating blood biomarkers were assayed using the following kits: Angiotensin II Enzyme-linked Immunosorbent Assay (ELISA) (E13652287, Sincere Biotech Co.,Ltd, New Taipei City 221, Taiwan (R.O.C.)); Cortisone ELISA (E13652408, Sincere Biotech Co.,Ltd, Sincere Biotech Co.,Ltd, New Taipei City 221, Taiwan (R.O.C.)); Architect Cortisol chemiluminescent microparticle immunoassay (CMIA) (8D15-25, Abbott Laboratories, Abbott Park, Illinois, USA); Liaison Aldosterone chemiluminescent immunoassay (CLIA) (310,450, DiaSorin, Sallugia, Italy); Innovance VWF:Ac (10,487,040/OPHL03, Siemens Healthcare Diagnostics, Marburg, Germany); and Tina-quant D-Dimer Gen.2 particle-enhanced immunoturbidimetric assay (04,912,551, Roche Diagnostics GmbH, Mannheim, Germany).

### Statistics

A blinded database was created in MS-EXCEL by the Delhi team. Two UK investigators assessed the data independently. Data was analysed with GraphPad Prism version 9. Following completion of data processing, the trial was un-blinded. To assess time to WHO OS ≤ 3, daily WHO OS were analysed by simple linear regression and two-way ANOVA (withdrawn patients who failed to reach WHO OS ≤ 3 were excluded from the analysis). Blood biomarkers were analysed via two-way ANOVA. Chi-square tests were applied to assess the difference between proportions for categorical variables, and the two-way ANOVA, Student’s t-test, and/or simple linear regression were used for assessing significance in the difference of means for continuous variables, as appropriate. The Mann–Whitney test was used where measurements followed non-Gaussian spread. A *p*-value < 0.05 was considered statistically significant.

### Registration

The trial was registered on the Clinical Trials Registry of India TRI: CTRI/2021/03/031721, reference: REF/2021/03/041472. Registered on 04/03/2021.

## Results

### Demographics

**Fig. 1:**
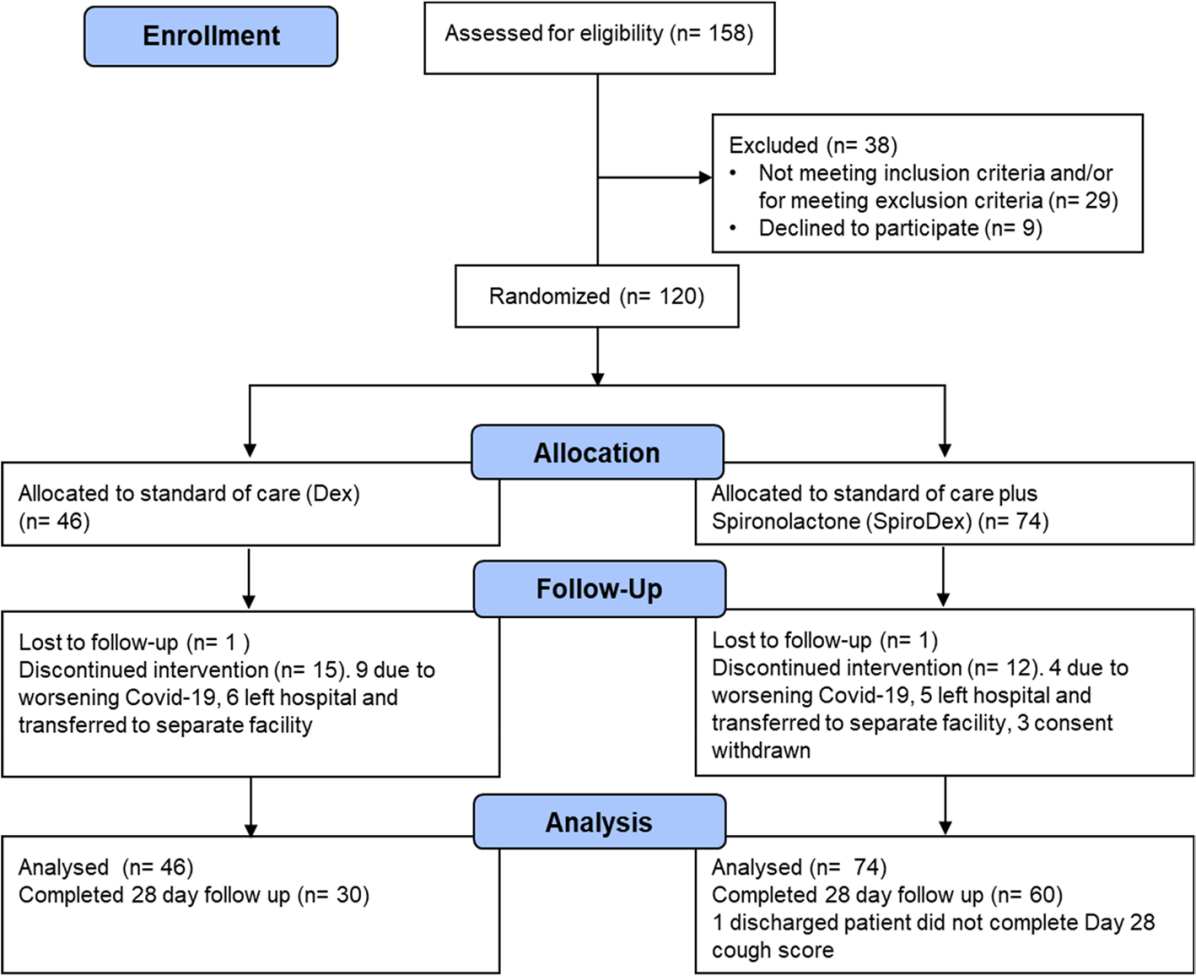
Consort diagram for both groups

A total of 158 hospital inpatients were screened for inclusion (Fig. 1). 38 were excluded due to not meeting inclusion criteria or patient choice. All 120 enrolled patients presented with acute symptoms of COVID-19 pneumonia, and were positive for SARS-CoV-2 by RT-PCR. 46 patients were randomised to receive standard of care (Dex), and 74 patients were randomised to receive Spironolactone (SpiroDex) in addition to standard of care.

The demographics for all patients are summarised in Table 1.

**Table 1:** Overview of patients enrolled to study and demographics at time of enrolment

	**Total** **(*n* = 120)**	**Standard of Care (Dex)** **(*n* = 46)**	**Standard of Care plus Spironolactone (SpiroDex)** **(*n* = 74)**
**Age (mean, [SD])**	48.8 [14.3]	49.1 [13.9]	48.6 [14.6]
**Gender**	Male: 61.7% (*n* = 74)	Male 67.4% (*n* = 31)	Male 58.1% (*n* = 43)
	Female: 38.3% (*n* = 46)	Female 32.6% (*n* = 15)	Female 41.9% (*n* = 31)
**Past Medical History**			
**Diabetes Mellitus**	34.2% (*n* = 41)	37.0% (*n* = 17)	32.4% (*n* = 24)
**Hypertension**	28.3% (*n* = 34)	21.7% (*n* = 10)	32.4% (*n* = 24)
**Coronary artery disease**	5.8% (*n* = 7)	8.7% (*n* = 4)	4.1% (*n* = 3)
**COPD**	1.7% (*n* = 2)	(*n* = 0)	2.7% (*n* = 2)
**Asthma**	3.3% (*n* = 4)	(*n* = 0)	5.4% (*n* = 4)
**Chronic Kidney Disease**	0.8% (*n* = 1)	(*n* = 0)	1.4% (*n* = 1)
**CVA**	0.8% (*n* = 1)	(*n* = 0)	1.4% (*n* = 1)
**Cancer**	0.8% (*n* = 1)	2.2% (*n* = 1)	(*n* = 0)
**Baseline Clinical Findings**			
**NEWS2 score Day 1**^**a**^** (median)**	3	4	3
**WHO OS score Day 1**^**a**^** (median)**	4	4	4
**ISARIC-4C score Day 1**^**a**^** (mean [SD])**	8.0 [3.1]	8.391 [3.09]	7.743 [3.11]
**SpO2/FiO2 ratio Day 1**^**a**^** (mean [SD])**	181.9 [61.1]	163.0 [58.3]	193.7 [60.3]
**D-Dimer μg/mL day 1**^**a**^** (mean [SD])**	1.35 [1.97]	1.798 [2.41]	1.062 [1.58]

^a^NEWS2: *p* = 0.0236 (Mann–Whitney). WHO OS: *p* = 0.5232 (Mann–Whitney). ISARIC 4C: *p* = 0.2684; 95% CI: -1.802 to 0.5061 (student’s t-test). SpO2/FiO2 ratio: *p* = 0.01; 95% CI: 6.846 to 50.18 (student’s t-test). D-Dimer: *p* = 0.047; 95% CI: 0.008769 to 1.463 (student’s t-test). NEWS2: National Early Warning Score 2. ISARIC-4C: a risk stratification tool that predicts in-hospital mortality for hospitalised COVID-19 patients. Score is calculated using parameters including age, co-morbidities and clinical biomarkers

6.8% (*n* = 5) of the SpiroDex arm were withdrawn as they voluntarily transferred to a separate hospital facility, compared to 13.0% (*n* = 6) of patients in the Dex arm. These patients have been included in the analysis up until transfer from the Lok Nayak facility. The clinical outcomes for these patients after withdrawal is unknown.

### Primary outcomes

**Fig. 2:**
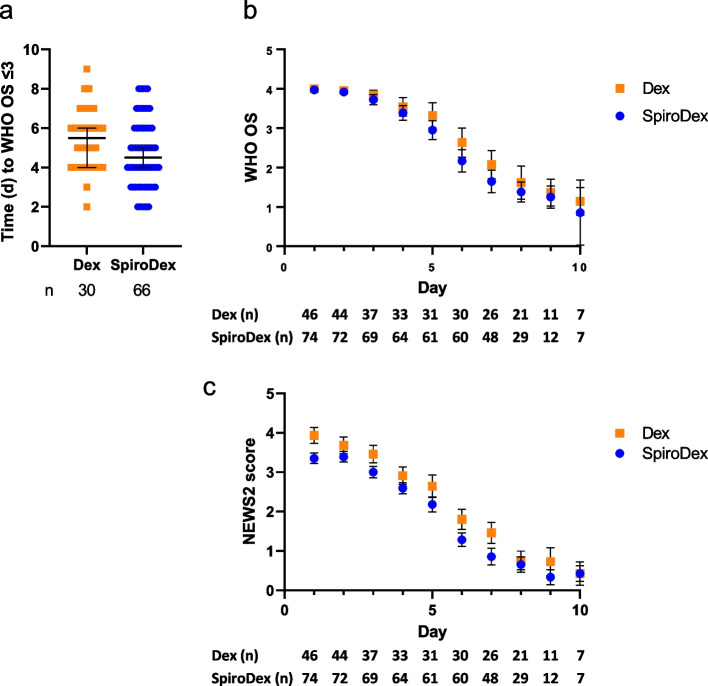
Primary Clinical Outcomes. **a** Time in days for patients to meet WHO OS ≤ 3. Median and 95% CI shown. Analysed by Mann–Whitney, *p* > 0.05. **b** Daily WHO OS measurement for all patients who met WHO ordinal scale ≤ 3. Mean and 95% CI shown. **c** Daily NEWS2 score for all patients. Mean and 95% CI shown. Patient number (n) included in analysis is shown below the plot. For all, Dex: squares, orange; SpiroDex: circles,blue.NEWS2: National Early Warning Score 2. WHO OS: World Health Organisation Ordinal Scale

**Fig. 3:**
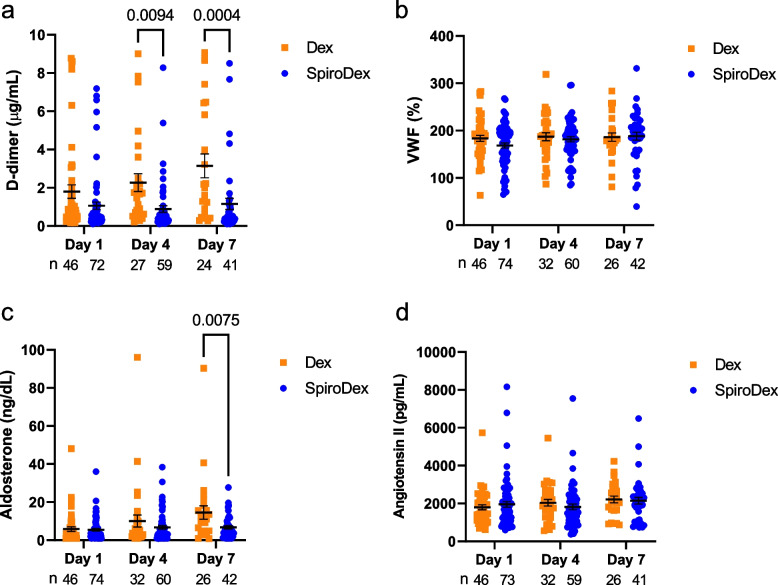
Primary Biomarker Outcomes. Day 1, 4 and 7 measurement of patient blood biomarkers (**a**) D-dimer, (**b**) VWF, (**c**) aldosterone and (**d**) angiotensin II. Mean and s.e.m shown. Analysed by two-way ANOVA, *p* < 0.05 indicated within the figure. For all, patient number (n) included in analysis is shown below the plot, Dex: squares, orange; SpiroDex: circles, blue

There were no statistical differences between the Dex and SpiroDex groups for the time in days for the patient to achieve WHO OS category 1, 2 or 3 (Fig. 2a). The Dex group had a median of 5.5 days, compared to a median of 4.5 days for the SpiroDex group (interquartile range (IQR) Dex 2.25 days, SpiroDex 3 days, *p* = 0.055). In addition, there was no significant difference in the rate of change for daily WHO OS or NEWS2 measurements for these patients (post hoc analysis) (Fig. 2b, c). The blood biomarker results demonstrated that there was significantly reduced plasma D-dimer in the SpiroDex treatment group at days 4 and 7 compared to the Dex group (Fig. 3a) (95% CI 0.77 to 3.2, *p* = 0.0004). VWF release was similar between both groups at days 1, 4 and 7 (Fig. 3b). Aldosterone levels were significantly lower in day 7 in the SpiroDex group compared to the Dex arm (95% CI 1.65 to 13.85, *p* = 0.0075) (Fig. 3c). Angiotensin II remained comparable across both groups (Fig. 3d).

### Secondary outcomes

**Fig. 4:**
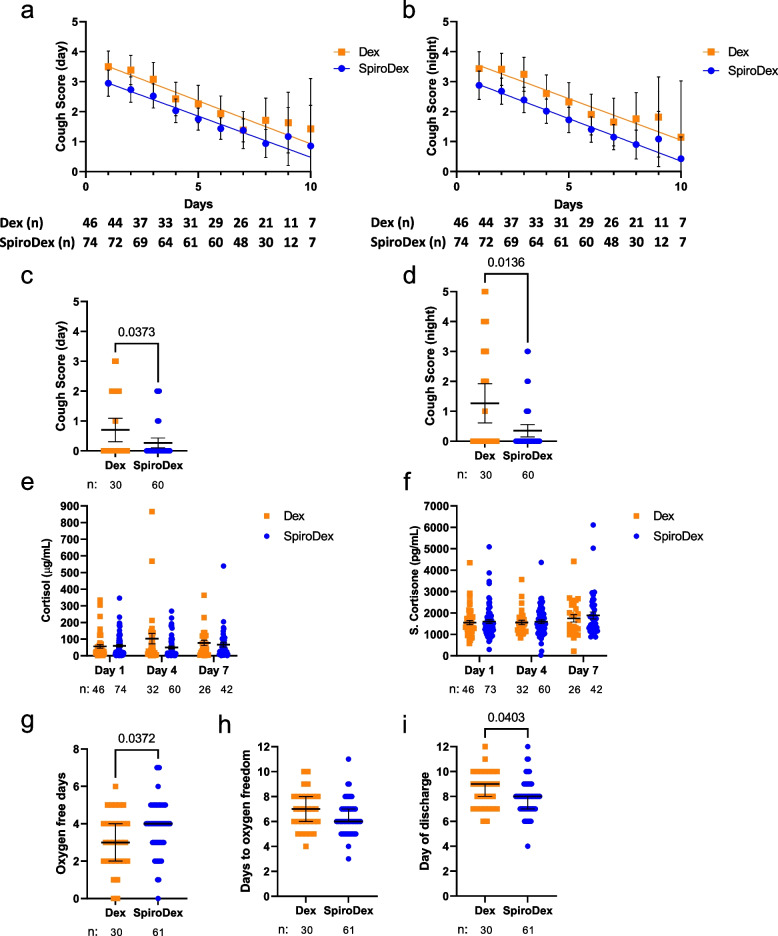
Secondary outcomes & post hoc analysis: Cough Score, oxygen use and plasma corticosteroid levels. **a** day-time and (**b**) night-time cough scores recorded on days 1–10. Range: 0 = no cough, 5 = distressing cough. Mean and 95% CI shown. Analysed by simple linear regression and two-way ANOVA. (**c**) Day 28 follow up day-time and (**d**) night-time cough score. Mean and 95% CI shown. Analysed by Mann–Whitney. Measurement of (**e**) plasma cortisol (**f**) and cortisone on days 1,4 and 7. Mean and s.e.m shown. Analysed by two-way ANOVA. **g** Supplemental oxygen free days, and (**h**) days to supplemental oxygen freedom. Median and 95% CI shown. Analysed by Mann–Whitney. **i** Days to patient discharge. Median and 95% CI shown. Analysed by Mann–Whitney. For all, patient number (n) included in analysis is shown below the plot, *p* < 0.05 indicated within the figure. Dex: squares, orange; SpiroDex: circles, blue

There was no significant difference in the cough scores during the acute illness between the two groups (Fig. 4a, b) however both day-time and night-time cough scores at day 28 were significantly lower in the SpiroDex group compared to the Dex group (Fig. 4c, d) (D28 day-time: *p* = 0.037, D28 night-time *p* = 0.0136). There were no significant differences in plasma corticosteroid levels between groups (Fig. 4e, f). The SpiroDex group had a median number of oxygen free days of 4, compared to the Dex group who had a median of 3 days (Fig. 4g) (*p* = 0.0372), and the patients in the SpiroDex group reached oxygen freedom one day sooner than the Dex group (median day 6 vs day 7, *p* = 0.06 (Fig. 4h).

### Post hoc analysis

82.4% (*n* = 61) of patients in the SpiroDex group were discharged from Lok Nayak Hospital (without reaching WHO OS scale > 4), compared to 65.2% (*n* = 30) of Dex patients. Of the patients who were discharged home, the SpiroDex patients were discharged earlier (median 8 days) than the Dex patients (median 9 days, *p* = 0.0403) (Fig. 4i). Fewer patients in the SpiroDex arm compared to the Dex arm (5.4% vs 19.6%) were removed from the trial due to worsening COVID-19 (pre-specified criteria of escalating to WHO OS > 4) (supplementary Fig. S1).

### Adverse events

Low dose spironolactone as an-add on therapy to standard of care was safe and well tolerated with no electrolyte differences between the groups (supplementary Fig. S2). There were no serious adverse events (SAEs) reported. All patients received the full dose of Spironolactone throughout their inclusion in the trial and no patient required a reduced dose due to impaired renal function. Other clinical and safety biomarkers were similar across both groups (supp Fig. S2). Aside from patients who were withdrawn due to worsening COVID-19 illness (Supplementary Table S1) /escalation to critical care, AEs recorded were mild to moderate and self-limiting (Supplementary Table S2).

## Discussion

In this randomised placebo-controlled trial, hospitalised patients who received low dose spironolactone and dexamethasone in addition to standard of care had significantly lower D-dimer levels at days 4 and 7, and a lower aldosterone level at day 7 in comparison with those on dexamethasone alone with standard care. We did not observe a significant reduction in the time to recovery to WHO OS ≤ 3 but did observe a significant increase in oxygen free days in the SpiroDex group, as well as a trend towards a shorter time to oxygen freedom and a significant reduction in day and night cough scores for SpiroDex patients at day 28.

Post hoc analysis demonstrated reduced clinical deterioration within the SpiroDex arm, and a reduced length of in-patient stay for those patients in the trial who were discharged from the Lok Nayak facility. In addition, treatment at this dose was safe with no recorded SAEs. This is the first report in a RCT setting of the potentially beneficial and safe addition of spironolactone to standard of care in COVID-19.

We note several limitations to this trial. There was a higher proportion of male patients in the Dex than the SpiroDex group and it is known that males are at a higher risk of severe illness in COVID-19 [16]. In addition, the SpiroDex group had statistically significant lower NEWS2 score and D-dimer levels and higher SpO2/FiO2 ratio at the time of recruitment (Table 1). The trial utilised manual randomisation with the inherent limitations that come with this method.

As the trial protocol did not allow for further data collection of patients after they deteriorated beyond WHO OS 4, the final outcomes for these individuals are unknown. In addition, a small number of patients had to be withdrawn from the trial due to their decision to be transferred to a different facility. In total, 12.2% of patients within the SpiroDex group withdrew from the trial versus 15.2% from the Dex group.

A raised D-dimer is associated with increased mortality in COVID-19 disease, and it is a recognised prognostic indicator of adverse outcomes [17, 18]. We observed a significantly reduced plasma D-dimer in the SpiroDex group at days 4 and 7 compared to the Dex group. This was also observed in a retrospective case series of MR antagonism [11], which reported a promising impact on mortality in patients with moderate to severe COVID-19, using intravenous canrenone, an MR antagonist. However, the dose of canrenone was dose equivalent to 120 mg per day of spironolactone, as compared to 25 mg used in our trial.

Loss of the specificity of the MR results in a key sequence of events. MR activation releases ATP which then acts on purinergic receptors on the cell and adjacent cells to increase intracellular calcium [8] and the exocytosis of Weibel Palade bodies from endothelial cells. These contain Von Willebrand Factor (VWF) and angiopoietin-II (A2). VWF promotes coagulopathy through interaction with platelets and A2 produces an increase in endothelial permeability. Both VWF and A2 are markedly elevated in patients severely ill with COVID-19 and are predictive of mortality [19–22]. Loss of specificity of the MR with consequent cortisol activation of the receptor results in suppression of aldosterone secretion. Using a highly specific aldosterone tandem mass spectrometric method (LCMSMS) assay it has been found that aldosterone levels were undetectable in a significant proportion of patients admitted to hospital with COVID-19 [23]. This is strong support for our basic hypothesis. Further support has come from recent studies on the purinergic system showing that there is a significant correlation between ATP, ADP and other purinergic metabolites with coagulation factors in COVID patients [24]. It should be noted that our aldosterone analysis was not performed with this advanced LCMSMS method, and we suggest further studies with spironolactone/dexamethasone combination therapies utilise this method for aldosterone analysis.

It is anticipated that SARS-CoV-2 will remain endemic within the global population even with a successful vaccination program. Together with the potential of variants of the SARS-CoV-2 emerging, new affordable and widely available treatments are needed. The promising safety biomarker (D-dimer) and clinical outcomes demonstrated in this phase 2 RCT support the evaluation of spironolactone together with dexamethasone in COVID-19 in larger randomised phase 3 controlled trials.

## Supplementary Information

**Additional file 1: **Supplementary information.** Table S1.** Further information on patients withdrawn from the trial due to worsening COVID19/ escalation to WHO Scale >4. **Table S2.** Summary of adverse events recorded for both groups. **Figure S1.** Kaplan Meier plot to show risk of patient escalating to WHO OS >4. **Figure S2.** Additional clinical biomarkers.

## Data Availability

All data produced in the present study are available upon reasonable request to the authors. Please contact the corresponding authors if required (BW, KD & CE).
