# Supplementary material for: Phase 2 randomised placebo-controlled trial of spironolactone and dexamethasone versus dexamethasone in COVID-19 hospitalised patients in Delhi
Source: BMC Infect Dis. 2023 May 15;23:326. doi: 10.1186/s12879-023-08286-w (PMC10184093; doi:10.1186/s12879-023-08286-w)
Supplement: Supplementary file 1 — Additional file 1: Supplementary information. Table S1. Further information on patients withdrawn from the trial due to worsening COVID19/ escalation to WHO Scale >4. Table S2. Summary of adverse events recorded for both groups. Figure S1. Kaplan Meier plot to show risk of patient escalating to WHO OS >4. Figure S2. Additional clinical biomarkers. [file 12879_2023_8286_MOESM1_ESM.docx]

**Supplemental data**

**Table S1** Further information on patients withdrawn from the trial due to worsening COVID19/ escalation to WHO Scale >4.

| **Patient ID:** | **Group:** | **Co-Morbid Conditions:** | **Clinical status prior to exclusion from study:** |
| --- | --- | --- | --- |
| 073 | Dex | None. | Increased Oxygen requirement on day 2, SpO2/FiO2 86, NEWS 9, WHO scale 5. On Bilevel positive airway pressure non invasive ventilation (BiPAP) |
| 053 | Dex | Pulmonary Tuberculosis, Diabetes Mellitus | Increased Oxygen requirement on day 3, SpO2/FiO2 80, NEWS 9, WHO scale 5, Shifted to BiPAP |
| 097 | Dex | Hypertension, Diabetes Mellitus | Increased Oxygen requirement on day 3, SpO2/FiO2 78, NEWS 9, WHO scale 5, Shifted to BiPAP |
| 002 | Dex | Thrombocytopenia, Diabetes, Hyponatremia, external Hemorrhoids, Lung cancer with liver and brain metastasis | Reduced GCS with seizures: Intubated and put on mechanical ventilation |
| 016 | Dex |  | Increasing Oxygen demand on day 2, SpO2/FiO2 87, NEWS 9, WHO scale 5, Shifted to BiPAP |
| 023 | Dex | Diabetes, Hypertension CAD (2017): EF 35%, Diabetes Mellitus, ex-Smoker. Alcohol excess. | Deteriorated on Day 4 with increased oxygen demand, SpO2/FiO2 90, NEWS 9, WHO scale 5, Shifted to BiPAP |
| 060 | Dex | None. | Deteriorated on Day 2 with increased oxygen demand, SpO2/FiO2 87, NEWS 8, WHO scale 5, Shifted to BiPAP |
| 062 | Dex | Diabetes Mellitus, SARI-T1 Respiratory Failure, CAD | Deteriorated on Day 2 with increased oxygen demand, SpO2/FiO2 89, NEWS 7, WHO scale 5, Shifted to BiPAP |
| 088 | Dex | SARI, T1RF, Hypertension, Diabetes Mellitus | Increased Oxygen requirement on day 6, SpO2/FiO2 90, NEWS 8, WHO scale 5, Shifted to BiPAP |
| 008 | SpiroDex | Chronic Cough, Liver Abscess | Increased Oxygen requirement on day 2, SpO2/FiO2 88, NEWS 8, WHO scale 5  Shifted to BiPAP ventilation |
| 010 | SpiroDex | Hepatitis, Abdominal Pain, jaundice, Ventriculomegaly | Intubated and put on mechanical ventilation in view of poor GCS and increasing oxygen requirement |
| 117 | SpiroDex | Hypertension, Diabetes,  Polio induced deformity | Increased Oxygen requirement on day 2, SpO2/FiO2 80, NEWS 9, WHO scale 5 put on BiPAP ventilation |
| 042 | SpiroDex | None. | Increased Oxygen requirement on day 2, SpO2/FiO2 78, NEWS 9, WHO scale 5, put on BiPAP |

**Table S2.** Summary of adverse events recorded for both groups

| **Adverse events recorded:** | | |
| --- | --- | --- |
| **Group:** | **# AEs** | **AEs:** |
| **Dex**: | 4 | 1. Abdominal pain, 2. Abdominal pain with cramping, 3. Constipation, 4. Worsening of COVID-19 (not requiring escalation to ITU or removal from trial) |
| **SpiroDex:** | 4 | 1. Body ache, 2. Nausea and vomiting, 3. Diarrhoea, 4. Syncope |

**Figure S1.** Kaplan Meier plot to show risk of patient escalating to WHO OS >4. Hazard ratio 0.21, 95% CI 0.070 to 0.64. For all, patient number (n) included in analysis is shown below the plot, p < 0.05 indicated within the figure. Dex: orange; SpiroDex: blue.

**Figure S2. Additional clinical biomarkers.**

Day 1, 4 and 7 measurement of patient blood biomarkers **(a)** sodium, **(b)** potassium **(c)** blood sugar, and **(d)** Urea. Data shows mean and SEM. Dex: squares, orange; SpiroDex: circles, blue.

**Utilising a manual coin flip method for 2:1 randomisation**

A coin toss follows binomial distribution (heads or tails). If a total of 6 coin tosses are considered, the probability of flipping ≥3 heads is 0.66, the therefore provides a 2:1 randomisation.

This can be proven with the following mathematical equation:

$$P(x)=\frac{n!}{x!\left( n-x \right)!}p^{x}q^{n-x}$$

Where:

*n* = total number of tosses

*x* = number of successes desired

! = factorial

*p* = probability of getting a success (head) in one trial

*q* = 1 – *p* = the probability of getting a failure (tail) in one trial

| **Probability of tossing ≥3 heads in 6 tosses** | | | | | | | |
| --- | --- | --- | --- | --- | --- | --- | --- |
| *n* = | 6 tosses | | 6 tosses | | | 6 tosses | 6 tosses |
| *x* = | 3 heads | | 4 heads | | | 5 heads | 6 heads |
| *p =* | 0.5 | | 0.5 | | | 0.5 | 0.5 |
| *q =* | 0.5 | | 0.5 | | | 0.5 | 0.5 |
|  |  | |  | | |  |  |
| *n*! = | 720 | | 720 | | | 720 | 720 |
| *x*! = | 6 | | 24 | | | 120 | 720 |
| (n-*x*)! | 6 | | 2 | | | 1 | 1 |
| *p^x^* | 0.125 | | 0.0625 | | | 0.03125 | 0.015625 |
| *q^n-x^* | 0.125 | | 0.25 | | | 0.5 | 1 |
|  |  | |  | | |  |  |
| **P(*x*) =** | 0.3125 | | 0.234375 | | | 0.09375 | 0.015625 |
|  |  | |  | | |  |  |
|  |  |  | |  |  | | |
| **Probability of tossing ≥3 heads = sumP(*x*) = 0.65625** | | | | | | | |
